# The prevalence of pulmonary hypertension assessed using the pulmonary vein‐to‐right pulmonary artery ratio and its association with survival in West Highland white terriers with canine idiopathic pulmonary fibrosis

**DOI:** 10.1186/s12917-021-02879-w

**Published:** 2021-04-23

**Authors:** Elodie Roels, Aline Fastrès, Anne-Christine Merveille, Géraldine Bolen, Erik Teske, Cécile Clercx, Kathleen Mc Entee

**Affiliations:** 1 Department of Internal Medicine, Pride Veterinary Centre, DE24 8HX Derby, United Kingdom; 2 Department of Clinical Sciences, Faculty of Veterinary Medicine, FARAH, University of Liege, 4000 Liege, Belgium; 3 Department of Clinical Sciences of Companion Animals, Faculty of Medicine, Utrecht University, 3584 CL Utrecht, The Netherlands; 4 Laboratory of Physiology and Pharmacology, Faculty of Medicine, Université Libre de Bruxelles, 1070 Brussels, Belgium

**Keywords:** Dog, Cardiac disease, Echocardiography, Pulmonary arterial pressure, Tricuspid regurgitation

## Abstract

**Background:**

Pulmonary hypertension (PH) is a known co-morbidity in West Highland white terriers (WHWTs) affected with canine idiopathic pulmonary fibrosis (CIPF). The pulmonary vein-to-right pulmonary artery ratio (PV/PA) has recently been described for the detection of pre-capillary PH in dogs. The objective of the present study was to estimate the prevalence of PH at diagnostic, in WHWTs affected with CIPF, by using PV/PA, in comparison with a group of healthy breed-matched controls (CTRLs). Additional study objective was to explore whether the presence of PH at initial diagnosis of CIPF impacted survival time in dogs treated with sildenafil.

**Results:**

Twenty-five client-owned WHWTs presented with CIPF and 19 CTRLs were included in the study. PV/PA in either two-dimensional mode (2D) or time-motion mode or both were measured from cineloops in each dog. Dogs were classified according to PV/PA value into non/mild PH (PV/PA measured in 2D ≥ 0.7) or moderate/severe PH (PV/PA < 0.7). Survival data of WHWTs affected with CIPF were extracted from medical record to assess association between presence of PH at diagnosis and outcome. 60 % overall prevalence for moderate/severe PH was estimated in this cohort of WHWTs presented with CIPF vs. 5 % in CTRLS (*P* = 0.0002). The presence of moderate/severe PH at initial presentation was not associated with survival.

**Conclusions:**

Results of the present study confirm a high prevalence of PH at diagnosis in WHWTs affected with CIPF and highlight the utility of PV/PA as a non-invasive surrogate for assessment of PH in this population.

## 1. Background

Canine idiopathic pulmonary fibrosis (CIPF) is a progressive fibrotic lung disease mainly reported in aged West Highland white terriers (WHWTs) [1, 2]. CIPF shares clinical, imaging and histopathological findings from both human idiopathic pulmonary fibrosis (IPF) and other interstitial lung diseases displaying non-specific interstitial pneumonia pattern [1–5]. In human IPF, pulmonary hypertension (PH) occurs in up to 85 % of patients, depending on disease severity, evaluation method and diagnostic criteria employed [6, 7]. The presence of PH in human IPF patient has been associated with poorer quality of life, reduced cardiopulmonary function and shorter survival time [8–10].

Right heart catheterization is considered as the gold standard method of diagnosing PH but is not routinely performed in clinical situations due to the invasiveness of the procedure. Doppler-echocardiography can be used to non-invasively assess pulmonary arterial pressures (PAP) in the presence of tricuspid or pulmonic regurgitation by applying the simplified Bernoulli equation [11]. However, in the absence of a tricuspid or pulmonic regurgitant jet, or in situation of poor Doppler alignment, this measurement may be not feasible or inaccurate [12, 13].

Among the other echocardiographic parameters currently available to non-invasively assess the presence of pre-capillary PH, the pulmonary vein-to-right pulmonary artery ratio (PV/PA) has recently been demonstrated to be one of the most accurate at predicting PAP estimated by peak tricuspid regurgitation systolic pressure gradient (TRPG) [14]. Indeed, PV/PA has been demonstrated to provide a good accuracy for the diagnosis of moderate to severe pre-capillary PH (defined as TRPG > 50mmHg) using a cutoff value of < 0.70 (sensitivity = 96 %, specificity = 82 %) [14].

To the author’s knowledge, there has been only one study, published over a decade ago, reporting an estimated prevalence for PH of more than 44 % in WHWTs affected with CIPF [15]. There has been no report studying the association between the presence of PH at diagnosis and survival in WHWTs affected with CIPF.

We hypothesized that PH occurs at a high prevalence rate in WHWTs affected with CIPF at initial diagnosis and has a negative impact on survival. The primary aim of this study was to estimate the prevalence of PH at diagnosis, in a cohort of WHWTs affected with CIPF, compared with a group of breed-matched healthy controls (CTRLs) using PV/PA echocardiographic parameter [14]. As a secondary aim, we explored whether the presence of PH at initial diagnosis of CIPF impacted survival time.

## 2. Results

### Dogs

Detailed features of the study population are summarized in Table 1. Forty-four WHWTs were included in the study: 25 WHWTs affected with CIPF and 19 CTRLs.

**Table 1:** Characteristics of the study population

	CIPF WHWTs (*n* = 25)	Control WHWTs (*n* = 19)	P-value
Sex, M/F	12/13	13/6	0.176
Age, years	10.6 +/- 2.5	8.5 +/- 2.8	0.009
Body weight, kg	9.6 (8.4–10.0)	9.0 (8.2–9.6)	0.255

CIPF, canine idiopathic pulmonary fibrosis; WHWTs, West Highland white terriers; M, male; F, female. Data are expressed as mean +/- standard deviation for normally distributed data and as median and interquartile range for not normally distributed data

WHWTs affected with CIPF were older than CTRLs, but there was no significant difference in gender repartition nor body weight between groups (Table 1). Two CIPF WHWTs had arrhythmia (one dog with sick sinus syndrome and another with second degree atrioventricular block), while none of the other dogs had significant rhythm abnormalities other than sinus arrhythmia. Among the 25 WHWTs affected with CIPF included, 17 (68 %) of them had a history of both exercise intolerance and cough, 5 (20 %) had exercise intolerance alone and 3 (12 %) dogs exhibited cough only. Crackles were noticed on lung auscultation in all CIPF WHWTs, restrictive dyspnea was present in 15 (60 %) dogs, 6 of them exhibiting additional cyanosis. At the time of initial echocardiography, treatments started by the referring veterinarian included prednisolone in 9 dogs (0.1–0.7 mg/kg q12-72 h PO), pimobendan in 3 dogs (0.2 mg/kg q12h PO), theophylline in 3 dogs (5–11 mg/kg q12h PO), furosemide in 2 dogs (2 mg/kg q12h PO), benazepril in 2 dogs (0.5–0.7 mg/kg q 24 h PO), spironolactone in 1 dog (4 mg/kg q24h PO), codeine in 1 dog (1 mg/kg q12h PO), sildenafil in 1 dog (0.6 mg/kg q24h PO) and antibiotics in 4 dogs. Thoracic high-resolution computed tomography (HRCT) was performed in 22/25 (88 %) WHWTs affected with CIPF and revealed extensive ground-glass opacity in all of them. Other thoracic HRCT findings included a combination of mosaic pattern, bronchial wall thickening, consolidation, parenchymal and subpleural bands, nodules and bronchiectasis. Thoracic X-rays images were available in the remaining 3 WHWTs affected with CIPF and displayed a severe diffuse broncho-interstitial pattern consistent with the CIPF diagnosis.

CTRLs WHWTs were clinically healthy and did not have any signs or findings indicating pulmonary or cardiovascular disease. None of them were receiving treatments. Thoracic HRCT was performed in 15/19 (79 %) CTRLs and did not reveal significant abnormalities, except for localized ground-glass opacity in cranial lung lobes in 1 of them which was attributed to mild degree of lung atelectasis. Among the 4 CTRLs without HRCT, one had a thoracic X-rays available without reported abnormalities. The echocardiographic images and the electrocardiogram were unremarkable in all dogs.

### Echocardiographic results

Echocardiographic data are presented in Table 2. Echocardiographic images for PV/PA measurements were obtained in both 2-dimensional mode (2D) and time-motion mode (MM) in 23/25 (92 %) CIPF WHWTs and all CTRLs. Two CIPF WHWTs had the PV/PA measured either in 2D or MM only (1 dog respectively), due to respiratory related artifacts. Using the cut-off value for PV/PA measured in 2D of < 0.7 corresponding to a TRPG > 50mmHg [14], moderate to severe PH was identified in 15/24 (63 %) WHWTs affected with CIPF and in 1/19 (5 %) CTRLs (*P* = 0.0002). The one WHWT affected with CIPF for which PV/PA (2D) was not measured had a PV/PA (MM) of 0.960 and a TRPG of 28.4mmHg, suggesting the absence of PH. Accordingly, the overall prevalence for moderate to severe PH in WHWTs affected with CIPF was estimated at 60 %. A tricuspid valve regurgitant jet was present in 12/25 (48 %) WHWTs affected with CIPF and in 5/19 (26 %) CTRLs (*P* = 0.14). TRPG measured in WHWTs affected with CIPF was in favor of none, mild or moderate PH in respectively 3, 6 and 3 CIPF WHWTs [16]. All WHWTs affected with CIPF with a TRPG indicative of moderate PH had a PV/PA measured in 2D < 0.7. TRPG measured in CTRLs indicated none or mild PH in 4 and 1 dog respectively; PV/PA measured in 2D was ≥ 0.7 in all of these dogs. A pulmonary valve regurgitant jet was present in 4/25 (16 %) CIPF WHWTs and 1/19 (5 %) CTRLs (*P* = 0.25). Only one of the CIPF affected WHWTs had a peak pulmonary regurgitation pressure gradient (PRPG) above 20mmHg, suggestive of an increase in mean PAP [16]. This dog had a PV/PA < 0.7.

**Table 2:** Echocardiographic results

	n	CIPF WHWTs	n	Control WHWTs	P-value
PV diam. (MM), mm	24	5.6 +/-1.6	19	7.6 +/- 1.6	< 0.001
PA diam. (MM), mm	24	9.0 +/- 0.8	19	7.5 +/- 0.8	< 0.001
PV/PA ratio (MM)	24	0.6 +/- 0.2	19	1.0 +/- 0.2	< 0.001
PV diam. (2D), mm	24	5.0 (3.9–5.6)	19	7.0 (6.1–7.3)	< 0.001
PA diam. (2D), mm	24	8.5 (7.4–9.0)	19	7.0 (6.6–7.7)	0.001
PV/PA ratio (2D)	24	0.6 (0.5–0.7)	19	1.0 (0.9–1.0)	< 0.001
TRPG, mmHg	12	41.2 +/- 16.2	5	26.2 +/- 15.4	0.100
PRPG, mmHg	4	14.0 +/- 10.2	1	4.8 +/- 0.0	0.477

CIPF, canine idiopathic pulmonary fibrosis; WHWT, West Highland whiter terrier; PV, right pulmonary vein; MM, time-motion mode; PA, right pulmonary artery; PV/PA, pulmonary vein to right pulmonary artery; 2D, 2-dimensional mode; TRPG, peak tricuspid regurgitation systolic pressure gradient; PRPG, peak pulmonary regurgitation diastolic pressure gradient. Data are expressed as mean +/- standard deviation for normally distributed data and as median and interquartile range for not normally distributed data

### Survival

**Fig. 1:**
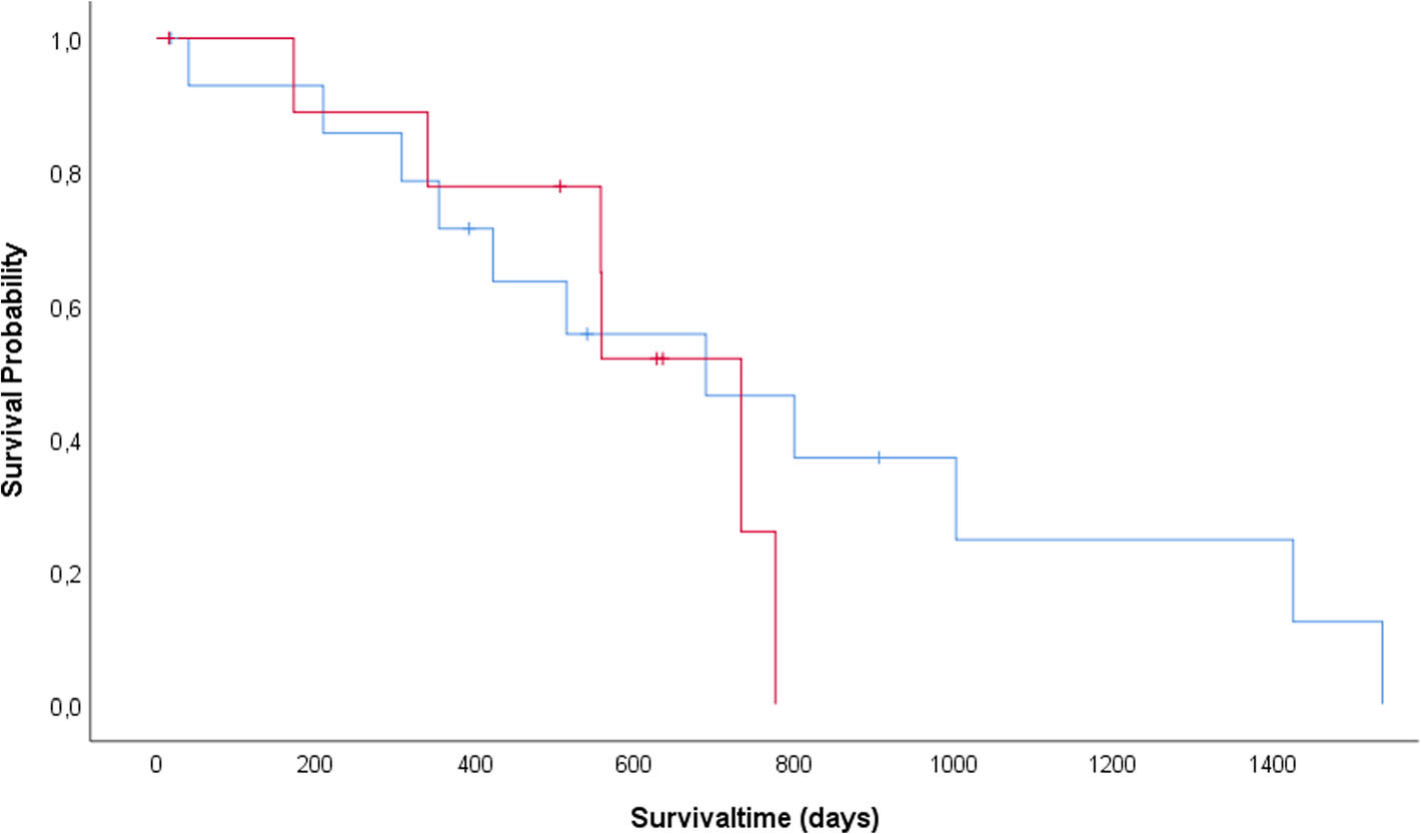
Survival time in CIPF WHWTs with moderate to severe and none to mild PH. Kaplan-Meier curves to compare survival time in days from canine idiopathic pulmonary fibrosis (CIPF) diagnosis (x-axis) between West Highland white terriers (WHWTs) affected with CIPF with moderate to severe pulmonary hypertension (PH) (group 1) (pulmonary veinto-right pulmonary artery ratio (PV/PA) measured in two-dimensional mode (2D) < 0.7) and WHWTs affected with CIPF with none to mild PH (group 2) (PV/PA measured in 2D ≥ 0.7). CIPF-related mortality: moderate to severe PH median survival, 698 days; none to mild PH median survival, 733 days. Log-rank P-value of 0.528

A total of 18 out of 25 WHWTs affected with CIPF died by the end of the study period, 4 dogs were still alive, and 3 dogs were lost of follow-up. The cause of death was CIPF-related in 17 dogs (94 %) with an overall median survival time of 557 days (40–1538 days). Lung tissue samples were available in 11 of these dogs (61 %) and allowed the histopathological confirmation of the CIPF diagnosis. There was no statistical difference in survival between WHWTs affected with CIPF having moderate to severe PH (median survival time 689 days; 95 % confidence interval (CI) = 260–1118 days) and WHWTs affected with CIPF having no or mild PH (median survival time 733 days; CI = 553–913 days) (*P* = 0.528, Fig. 1) at diagnosis. Univariate Cox-regression analysis revealed no significant effect of PV/PA measured in both modes on survival (hazard ration of 5.98 (CI = 0.36–100.80) and *P* = 0.198 for PV/PA measured in 2D; hazard ration of 3.78 (CI = 0.18–81.00) and *P* = 0.385 for PV/PA measured in MM).

## 3. Discussion

Assessment of PH can be challenging in veterinary medicine as Doppler estimation of PAP via measurement of TRPG is not always feasible or accurate [13]. This is particularly true in WHWTs affected with CIPF as they are frequently presented with laborious breathing that can impair proper Doppler beam alignment. In this study, we used the newly described PV/PA echocardiographic parameter as a surrogate diagnostic tool for pre-capillary PH [14]. We estimated a 60 % overall prevalence for moderate to severe PH in our cohort of 25 WHWTs affected with CIPF by using this parameter. The presence of moderate to severe PH at initial presentation in this group of dogs was not associated with a shorter survival.

After publication of PV/PA reference intervals in healthy dogs [17, 18] and of its use for prediction of congestive heart failure in mitral valve disease [18], our team recently described the use of PV/PA (measured in 2D) to predict moderate to severe pre-capillary PH (TRPG > 50 mmHg) [14]. PVPA was proven to predict PH with a good diagnostic accuracy (AUC 0.94) when compared with other non-invasive echocardiographic parameters of PH [14]. Applying the reported cut-off value of < 0.7 for PV/PA measured in 2D [14], the overall prevalence for at least moderate PH was estimated at 60 % in the presently studied CIPF population. This finding is in agreement with human IPF literature reporting a PH prevalence ranging from 32 to 85 % according to studies [7]. This also corroborated previously reported prevalence of > 44 % for at least mild PH (according to TRPG value) in a cohort of 45 WHWTs affected with CIPF [15].

Using the same PV/PA cut-off value on a breed-matched healthy CTRLs population, one out of the 19 included dogs (5 %) was classified as suffering from moderate to severe PH. As this dog was clinically healthy, this result most likely indicates a false-positive and reflects the lack of specificity of the PV/PA parameter employed, previously reported at 82 % [14]. Another healthy CTRL included in the present study was found with a TRPG gradient indicative of mild PH. Although Doppler measurement of TRPG generally tends to underestimate PAP, overestimation is possible as demonstrated by Soydan and associates (2015) [13]. Another less likely explanation can be that these CTRLs dogs were suffering, despite normal HRCT findings, from subclinical early CIPF lesions at the time of echocardiographic examination causing PH. Histopathological examination of lung tissue and/or follow-up at regular intervals would have been needed to answer this issue, but was not performed.

A mean/median value of 1 for PV/PA measured in both modes was found in healthy CTRLs included in this study, as previously reported in healthy dogs from other breeds [17, 18]. Significantly lower PV/PA values were observed in WHWTs affected with CIPF compared with CTRLs, attributed to both an increase in the right pulmonary artery (PA) size and a decrease in the right pulmonary vein (PV) size (Table 2). This finding corroborates previously reported observations in other causes of pre-capillary PH, notably angiostrongylosis and bronchomalacia [14]. Enlarged PA size was previously discussed as a possible consequence of increased pulmonary vascular bed resistance due to the underlying lung disease, while decreased PV size was speculated to stem from reduction of left ventricular preload secondary to increased PAP, from decrease right ventricular function, from compression of the vein by the enlarged adjacent artery, or some combination of these factors [14].

Surprisingly, the presence of moderate to severe PH at initial echocardiographic examination was not associated with a shorter survival in our CIPF cohort. This finding contrasts with the human literature where IPF patients having PH had a significantly increased 1-year mortality rate in comparison with IPF patients without PH (28 % vs. 5.5 %) [19]. Another study also reported a three-fold increase in mortality in IPF patients with PH, especially when systolic PAP exceeds 50mmHg at echocardiography [20]. This finding in CIPF dogs also differs from a recent canine study about PH secondary to respiratory disease, mainly obstructive airway/lung disease (81 % of included dogs), where dogs with an estimated PAP ≥ 47mmHg had significantly shorter survival than those < 47mmHg [21]. The most likely explanation for the lack of association between survival and PH in our cohort of WHWTs affected with CIPF is that all WHWTs affected with CIPF suffering from PH at diagnosis were subsequently treated with sildenafil and then regularly followed as part of the CIPF-project to adjust treatment recommendations. Sildenafil treatment in pre-capillary PH has been shown to lower PAP from baseline, improve quality of life, and expand likelihood of survival in dogs [21–23]. Accordingly, sildenafil administration in our cohort of CIPF WHWTs affected with pre-capillary PH could have contributed to improved survival and prevented identification of an association between the presence of PH at diagnosis and outcome. Another explanation for the lack of association with survival is that the natural course of CIPF has been shown to vary greatly from one dog to another, with WHWTs presenting either a rapid or a slow disease progression [24]. Whether the different types of disease progression are influenced by underlying comorbidities such as PH is currently unknow. Lastly, CIPF in dogs is not exactly the same disease as IPF in humans as tomographic and histopathologic findings in CIPF have been shown to cover both IPF and non-specific interstitial pneumonia pattern characteristics [1–5]. Accordingly, the association between PH and survival can be different in CIPF than in human IPF or than in other canine respiratory conditions.

The main limitation of the present study was that PAP were not directly measured by right heart catheterization to assess the exact prevalence of mild, moderate and severe PH at CIPF diagnosis. This technique was not chosen because it is invasive, has inherent risk of complications and would have required heavy sedation or general anesthesia. Instead, PV/PA was chosen over TRPG to estimate the presence of PH, as TRPG was unmeasurable in a substantial number of dogs which would have led to an underestimation of the prevalence of PH. The second limitation of the present study was that a small proportion of WHWTs affected with CIPF included were receiving cardiac or vasoactive drug therapies at the time of echocardiography which could have interfered with PV/PA measurements (e.g. diuretics potentially reducing the PV size vs. sildenafil or pimobendan potentially reducing the PA size). Two CIPF WHWTs also had arrhythmias at the time of echocardiographic examination which could have impacted vessels size and PV/PA measure. The small population size was a third limitation, however given the suspected low prevalence of CIPF in the WHWT breed population, this patient cohort can be considered as relevant. Three CTRL WHWTs did not have any imaging performed to confirm the absence of pulmonary lesions. Pulmonary fibrosis was unlikely in these dogs as they were not displaying any clinical signs of cardiorespiratory disease. Lastly, echocardiographic parameters were assessed at diagnosis in all included dogs to estimate prevalence of PH at study inclusion. A follow-up study to assess the incidence of PH in WHWTs affected with CIPF over disease progression would have been interesting, but was not performed in the present study.

## 4. Conclusions

Results of the present study revealed an estimated overall prevalence of 60 % for moderate to severe PH at diagnosis of CIPF as defined by the PV/PA echocardiographic parameter. This high prevalence rate for PH at diagnosis should encourage practitioner to perform echocardiography on a regular basis in WHWTs presented with suspicion of CIPF. The presence of PH at diagnosis was not associated with survival in WHWTs affected with CIPF. Further studies are needed to determine the utility of PV/PA in the long-term monitoring of WHWTs affected with CIPF for assessing the incidence of PH development in the course of the disease or assessing the effects of targeted therapy.

## 5. Methods

### Dogs

In order to estimate the PH prevalence at diagnosis using PV/PA, in WHWTs affected with CIPF compared with a group of breed-matched healthy controls and explored whether the presence of PH at diagnosis of CIPF impacted survival time, client-owned WHWTs presented with CIPF and CTRLs were enrolled at the Small Animal Veterinary Clinic of the University of Liège under the umbrella of the CIPF project^1^ between April 2012 and November 2018 with owner’s consent.

Dogs were retrospectively included in the present study if PV/PA was recorded either on 2D or MM or both during initial echocardiographic examination. Dogs with a concomitant left- or right-sided cardiac disease were excluded.

WHWTs affected with CIPF were diagnosed according to a previously described approach [25]. CIPF diagnosis was based on history, presence of crackles on lung auscultation, and compatible thoracic imaging findings. Diagnosis was further confirmed by lung histopathology for dogs who died during the study period and for which lung tissue was available. WHWTs affected with CIPF were subdivided into two groups according to PV/PA echocardiographic evidence of pre-capillary PH: non/mild PH group (PV/PA measured in 2D ≥ 0.7) and moderate/severe PH group (PV/PA measured in 2D < 0.7).

Breed-matched control dogs were recruited if healthy with no previous history of cardiovascular or pulmonary disease and normal general and cardiopulmonary physical examination. All dogs recruited under the umbrella of the CIPF project had an echocardiographic study performed as part of their initial investigation.

### Echocardiographic examination

Transthoracic 2D, MM and conventional Doppler-echocardiography were performed by three trained observers including two board-certified veterinary cardiologists (KME and ACM) and one cardiology assistant under the direct supervision of a board-certified veterinary cardiologist, using an ultrasound unit (Vivid I, General Electric Medical System, Machelen, Belgium) equipped with 2.2–3.5 and 5.5–7.5 MHz phased-array transducers. Dogs were placed in right and left lateral recumbency and a simultaneous one-lead electrocardiogram was recorded. Standard right parasternal (long and short axis) and left apical parasternal views were used for echocardiographic data acquisition.

Echocardiographic measurements were retrospectively performed off-line by a single trained investigator (KME). As previously described [14, 17, 18] and illustrated [14], a right parasternal long axis four-chamber view was optimized to simultaneously record both PV and PA vessels. Dimensions of both vessels were obtained as previously described [14]. An average of at least 3 representative measurements of PV and PA diameters were taken in MM and 2D to calculate PV/PA ratios [14, 18]. Peak systolic tricuspid regurgitant jet velocity, when present, was measured using continuous wave Doppler from the view that allowed the best alignment to calculate TRPG. The highest value of tricuspid regurgitant jet velocity was recorded. A similar technique was used to record pulmonary regurgitation, when present, using continuous wave Doppler positioned centrally in the flow stream between the opened pulmonic valve leaflets and measure the PRPG.

### Survival analysis

Outcome data were obtained from clinical records for WHWTs affected with CIPF. Death was defined as CIPF-related if dogs were euthanized or died due to clinical signs of progressive respiratory failure (dyspnea, cyanosis, severe cough, extreme fatigability, syncope, etc.). The decision to euthanize was made by the owners if dog’s quality of life could not be maintained. Euthanasia were performed in a standard manner using pre-medication with butorphanol 0.3 mg/kg IV, anesthesia induction with propofol 1 mg/kg IV and final lethal dose of pentobarbital 200 mg/kg IV. Cardiopulmonary arrest was confirmed by careful and prolonged cardiac auscultation.

### Statistical analysis

Statistical analyses were performed using XLstat (Addinsoft SARL, Paris, France) and SPSS for Windows v25.0 (IBM Corp, Armonk, New York).

Continuous variables were reported as either mean +/- standard deviation for normally distributed data or median and interquartile range for not normally distributed data, and categorical data as proportions. The Shapiro-Wilk test was applied to assess the normality of distribution of continuous variables. Differences in continuous variables among CIPF and CTRLs WHWTs were determined using student’s t-test (for normally distributed variables) or Mann-Whitney test (for variables that were not normally distributed). Proportions were compared between groups using the Chi-squared test. Survival curves in days after CIPF diagnosis were drawn with the Kaplan-Meier method. Dogs that were still alive on the last date of follow-up or dead for another reason than CIPF were censored. Dogs lost to follow-up were included until the last day of known follow-up, at which point they were censored. Univariate tests for comparison of groups of survival data were made with the log-rank test and with a proportional hazard logistic regression model for continuous data. For all analyses, *P-*value ≤ 0.05 was considered statistically significant.

## Data Availability

The datasets used during the current study are available from the corresponding author on reasonable request

## Abbreviations

2D: two-dimensional mode
CI: 95% confidence interval
CIPF: canine idiopathic pulmonary fibrosis
CTRLs: controls
HRCT: high resolution computed tomography
IPF: idiopathic pulmonary fibrosis
MM: time-motion mode
PA: pulmonary artery
PAP: pulmonary arterial pressure
PH: pulmonary hypertension
PRPG: peak pulmonary regurgitation diastolic pressure gradient
PV: pulmonary vein
PV/PA: pulmonary vein-to-right pulmonary artery ratio
TRPG: peak tricuspid regurgitation systolic pressure gradient
WHWT: West Highland white terrier

## Footnotes

1 See: http://www.canineidiopathicpulmonaryfibrosis.ulg.ac.be.
